# Effect of nitrogen-doping configuration in graphene on the oxygen reduction reaction†

**DOI:** 10.1039/c8ra08576e

**Published:** 2019-02-19

**Authors:** Shih-Hsuan Tai, Bor Kae Chang

**Affiliations:** Department of Chemical & Materials Engineering, National Central University Zhongli District Taoyuan City 32001 Taiwan Republic of China BKChang@ncu.edu.tw

## Abstract

In this study, we investigate the oxygen reduction reaction (ORR) reactivity of nitrogen-doped graphene by using density functional theory (DFT), a computational quantum mechanical technique. Four doping configurations and five models were comprehensively studied: quaternary nitrogen (NQ), pyrrolic nitrogen (N5), two forms of pyridinic nitrogen (N6, N6nH) and three-pyridinic nitrogen (3N6). Models for possible sites during each step of ORR were set up and visualized to provide a platform to calculate the free energy of the reaction pathway to determine the suitability of each doping scenario. Associative mechanisms were displayed by all models except N5, which showed dissociative mechanism. The calculated free energy pathways demonstrate that the ranking of the reactivity for ORR by different nitrogen configurations from most reactive to least reactive is N6, NQ, N6nH, 3N6, and N5. Spin density and charge density aid in describing levels of reactivity.

## Abstract 2

The oxygen reduction reaction (ORR) reactivity of various nitrogen-doped graphene configurations are probed in detail using density functional theory (DFT) calculations.

## Graphical abstract

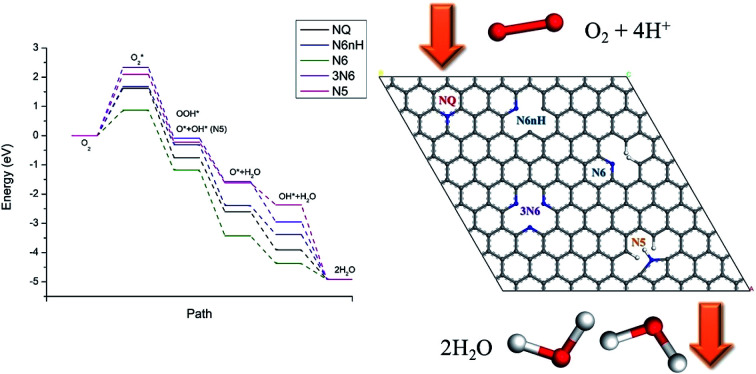

## Introduction

In the past few decades, fuel cells have caught the attention of scientists due to their stable performance in providing electrical power for extended periods through continuously supplying a source of fuel and air. Although still not prevalent in the whole world as a main method of energy storage, there are several applications of fuel cells to deal with specialized applications. For example, industries use it to generate electrical power, the military embed it into equipment to reduce weight, and car companies use it as a power generator to produce power for vehicles.

Fuel cells can directly convert chemical energy from a fuel into electricity with high power density, efficiency and in a more environmentally friendly fashion. The oxygen reduction reaction (ORR) is the main reaction on the cathode of fuel cells, and this reaction is limited by its slow kinetics, which in turn decides the overall performance of fuel cells.^1^ Traditionally, metallic materials such as platinum and its alloys are used at the cathode. However, due to the scarcity and price of these metals, non-metallic materials such as carbon nanotubes and nitrogen-doped graphene (NG) have been extensively studied both experimentally and theoretically to replace it in this field.^2–5^ Graphene and its derivatives are helpful for electrocatalytical application in fuel cells because of their electronic properties.

Graphene is a single layer of carbon atoms arranged in a hexagonal lattice or so-called honeycomb-like structure that was isolated from bulk graphite and further characterized in 2004.^6^ The material possesses properties such as unique electron and phonon structures, biological compatibility, delocalized π bonds, and controllable atomic thickness. These properties make graphene capable of having amazing potential in various fields in recent years. For example, it can be applied in biological engineering,^7^ energy storage,^5^ solar cells,^8^ sensing devices,^9,10^ and fuel cells.^11,12^

Doping graphene and carbon materials with heteroatoms has emerged rapidly because it can tailor the electronic properties of the resulting material to tune their chemical activity and to provide new possibilities for the application of such carbon materials. Nitrogen and boron are widely used to dope graphene for modification due to their atomic sizes, which are closer to that of carbon atoms. However, continuing efforts have also studied other elements for doping such as phosphorous, sulfur, halogen group atoms, iron, and co-doping in many fields.^13–20^

By now, it has been reported, both experimentally and computationally, that NG and carbon defects can facilitate ORR on the cathode in fuel cells. Nitrogen-doped carbon materials have shown excellent performance in this regard. However, the potential role of different nitrogen configurations in enhancing ORR is still under debate. Currently, the discourse is on whether active sites are formed by pyridinic nitrogen or graphitic nitrogen.^2,4,21–24^ Guo *et al.* considered this discrepancy was mainly due to two reasons.^24^ One is the mixing of different types of nitrogen configurations in carbon materials, causing indirect evidence to prove which configuration had the more active contribution. The other cause is associated with the morphology and graphitization level, which lead to inhomogeneous sizes of the π-conjugation system. She *et al.* reported very recently on the three major types of NG (pyridinic, graphitic or so-called quaternary, pyrrolic nitrogen) in both experimental and theoretical methods. Their experimental results showed that N6 was most ORR catalytically active, while their calculation results further asserted that graphene cluster with N6 possessed the largest number of active sites, *i.e.* carbon atoms with positive spin density values. However, they did not calculate the free energy pathway diagram for ORR which can provide a direct comparison of the ORR activity.^4^ Several reports have computationally investigated the ORR mechanism on several carbon materials such as graphene nanoribbons,^25^ tin-doped graphene,^26^ nitrogen-doped graphene cluster,^27^ or hydrogen-substituted graphdiyne (HsGDY).^28^ However, there is a lack in literature reporting a systematic study of NG with three-dimensional periodic boundary condition, which enables the computational system under study to emulate bulk graphene material properties closer to experimental observations, with different nitrogen configurations and discussion of ORR mechanism in theoretical aspects.

## Methods

All calculation of geometry optimization and electronic properties were implemented in CASTEP package^29^ with GGA-PBE functional^30^ and ultrasoft pseudopotentials, 560 eV plane wave basis set cutoff energy, and 2 × 2 × 1 Monkhorst–Pack *k*-point grid,^31^ at the spin unrestricted DFT-D level using the Grimme 2006 method.^32^ Geometry optimization convergence stopping criteria for change in energy, maximum force, maximum displacement, and maximum stress are 5 × 10^−6^ eV per atom, 0.01 eV Å^−1^, 5 × 10^−4^ Å, and 0.02 GPa, respectively.

**Fig. 1:**
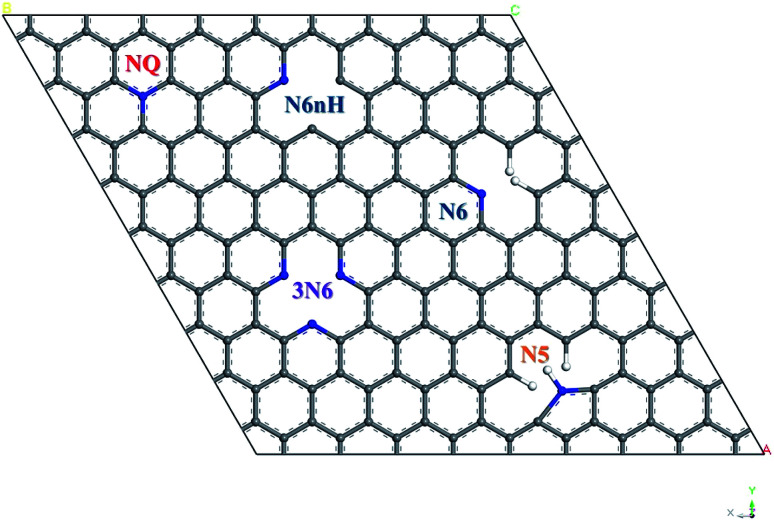
Schematic diagram of five models of nitrogen doped graphene.

To construct the graphene model, a unit cell containing two carbon atoms with 120° bond angle and lattice parameter of 2.46 Å was used, which is close to the experimental value of graphite from literature.^33^ This work used a 7 × 7 graphene supercell containing 98 carbon atoms. A vacuum layer of 12 Å was included to avoid artificial interactions between graphene layers, and periodic boundary condition in three dimensions was applied. Fig. 1 shows four types of nitrogen doping in graphene with five representative models: quaternary (NQ), pyrrolic (N5), pyridinic (N6, N6nH), and three-pyridinic (3N6). N5 and N6 were modeled as defects on the edge of graphene by adding hydrogen on the carbon near the nitrogen defect. N6nH on the other hand was placed inside the graphene material based on description in literature,^34^ and thus required no passivation.

**Scheme 1:**
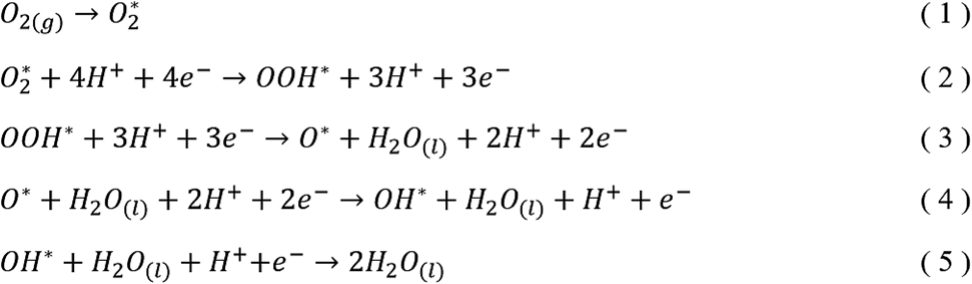
Associative mechanisms of ORR pathway in acidic media.

In this study, we calculated free energy diagrams of ORR on various NG model constructed from the total energy of each reaction pathway step shown in Scheme 1. First step of the reaction pathway was the adsorption of oxygen and the remaining four steps involved the addition of one hydrogen atom to the system during each step to form OOH*, O* + H_2_O, OH* + H_2_O, and 2H_2_O, respectively. The four electron associative mechanism has been reported as the favored ORR process in NG materials.^3,35^ In this work, the reference electrode was set to the NHE and *U* = 0 V and at the equilibrium potential *U* = 1.23 V, both at pH = 0. We calculated the free energy changes of the ORR due to the effects of various electrode potential conditions applied from the following equation:Δ*G* = Δ*E* + ΔZPE − *T*Δ*S* + Δ*G*_U_ + Δ*G*_pH_ + Δ*G*_solvation_where Δ*E* is the change of the total reaction energy based on our calculation, Δ*G*_U_ = −*neU*, where *n* is the number of transferred electrons and *U* is the electrode potential, and Δ*G*_pH_ = *k*_B_*T* × ln 10 × pH, where pH = 0. With free energy calculations based on Scheme 1, we can obtain energies of all terms in the equation except H_2_O_(l)_, O_2(g)_, and H^+^ from DFT calculations. Nonetheless, the free energy of H_2_O_(l)_ can be derived from 
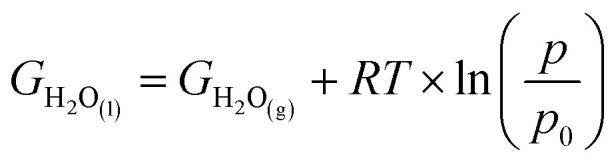
 where *T* = 298.15 K, *p* = 0.035 bar, *p*_0_ = 1 bar, the free energy of O_2_ can be derived from *G*_O_2__ = 2*G*_H_2_O_(l)__ − 2*G*_H_2__ + 4.92 eV, and the free energy of H^+^ can be calculated by 
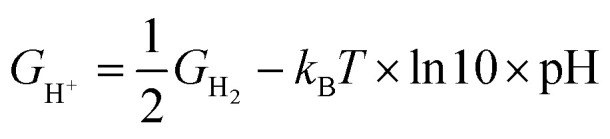
.

The solvation free energy correction was implemented using the APBS software since our models and calculations are under vacuum condition.^36–39^ Thus, the total energy was represented as the sum of gas-phase and solvation free energies:*E*_total_ = *E*_gas_ + *E*_solvation_

The parameters were set to the solvent-accessible surface for a solvent with a spherical radius of 1.4 Å and a dielectric constant of 78.54. The periodic boundary condition is not compatible with the APBS environment, we thus removed the lattice periodicity and passivated the structure for this set of calculations.

## Results & discussion

The five steps of the ORR pathway are shown in Scheme 1, which outlines the series of total energies calculated to emulate this reaction mechanism. There are four steps of the pathway that needed to be calculated explicitly, since by the fifth step the final structure is considered to have recovered to the same as the initial stage. Each step is denoted in sequential order by S1, S2, S3, and S4. In the case of the first step, the arriving oxygen molecule can be situated in various orientations on the graphene surface, where the two oxygen atoms might be on top of the nitrogen atom, carbon atoms, the hollow valley in the center of carbon rings, and any bond (or bridge). We use N (nitrogen), C (carbon), H (hollow), and B (bridge) to denote these possibilities for the first step, *i.e.* S1_CBC indicates that the oxygen molecule can be found across a bridge with each oxygen on top of carbon atoms. However, only one adsorption scenario is most likely to take place, and that should be the one with the lowest total energy and therefore the most stable configuration. Therefore, each of the above postulated models were geometry optimized to determine the correct energy and atomic arrangement for the first step of the ORR. For the remaining steps where hydrogen atom is added one at a time, we continue to denote S as step, and site as the position, *e.g.* the first position of the second step of NQ is mentioned as NQ_S2_site1.

**Fig. 2:**
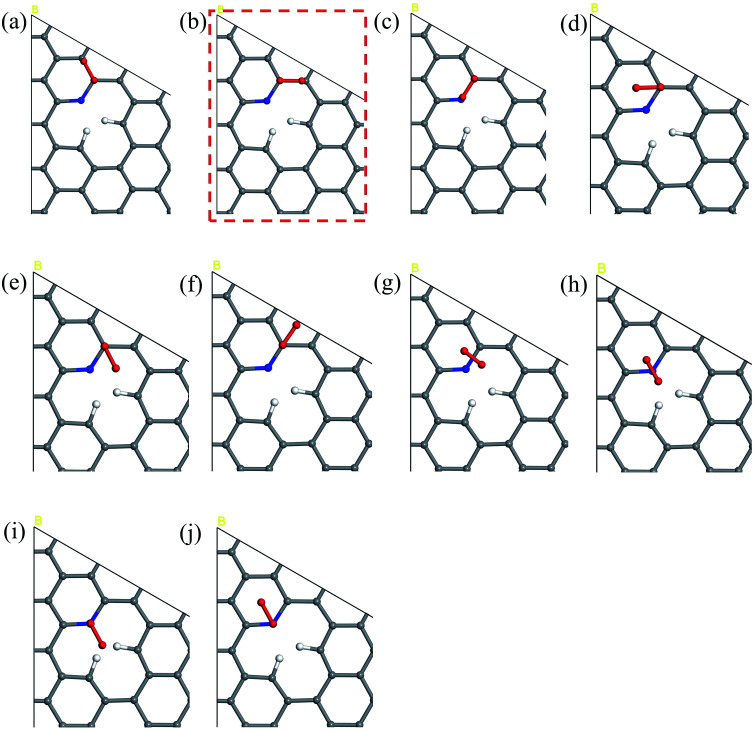
The potential sites where O_2_ can be located on the N6 substrate for the first step of the ORR. N6_S1_(a) CBC1 (b) CBC2 (c) CBN (d) CH1 (e) CH_2_ (f) CH_3_ (g) HBH (h) HNH (i) NH1 and (j) NH_2_. The model marked by red dashed line has the lowest energy upon geometry optimization, indicating the most likely adsorption configuration and is used in the free energy diagram.

**Fig. 3:**
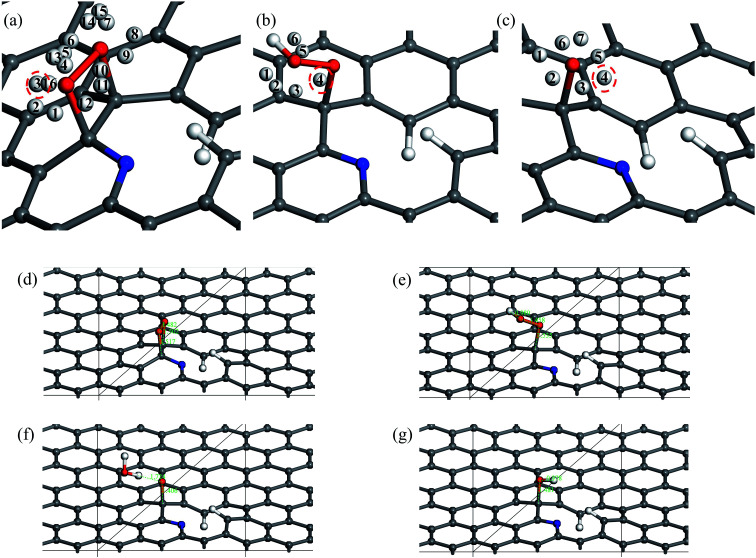
(a)–(c) Chosen sites for hydrogen atom placement on N6 substrate at steps 2–4 of the ORR. The three positions marked by red dash line are the lowest energy configuration and used in the free energy diagram. Structures with the lowest energy for steps 1–4 are shown in detail: (d) N6_S1_CBC2 (e) N6_S2_site3 (f) N6_S3_site4 (g) N6_S4_site4.

The 10 oxygen adsorption configurations for the first ORR step on N6 substrate are shown in Fig. 2. N6_S1_CBC2 has the lowest energy after optimization and was used in the subsequent step. For each of the following three steps, we added a single hydrogen atom with a distance of 0.9 Å from the oxygen molecule, which was close enough for bond formation, at different sites on the substrate shown in Fig. 3a–c to consider all possible adsorption sites. Each of these scenarios were optimized so that we could identify the structure with the lowest energy. In the case of the second step, the hydrogen atom placed at N6_S2_site3 (Fig. 3e) had the lowest energy and served as the starting point for the next set of calculations. Using this method, structures for step three were created by placing the hydrogen atom with a distance of 0.9 Å from the OOH complex, as shown in Fig. 3b, and revealed N6_S3_site4 as the lowest energy site (Fig. 3f). Finally, the fourth step where the final arriving hydrogen atom also has a distance of 0.9 Å from the oxygen atom (Fig. 3c) results in the lowest energy position N6_S4_site4 shown in Fig. 3g. The same technique was applied to the other nitrogen-doped models and details are described in the ESI.†

N5 was the only case to show a dissociative rather than associative mechanism. All the chosen sites for S2 (O_2_* + H^+^) ended up in the configuration of O* + OH* rather than OOH*. Therefore, we can conclude that the ORR on N5 prefers the dissociative mechanism, in stark contrast to the reaction pathway of NQ. By step 3 the two configurations have different adsorbed species on the graphene surface, therefore in NQ hydrogen atoms were added to the surrounding of the non-adsorbed molecular oxygen (Fig. S2b†), whereas in the N5 system they were added in the vicinity of the OH* (Fig. S4b†). All of the chosen sites and final structures with the lowest energies are detailed in the ESI.†

**Fig. 4:**
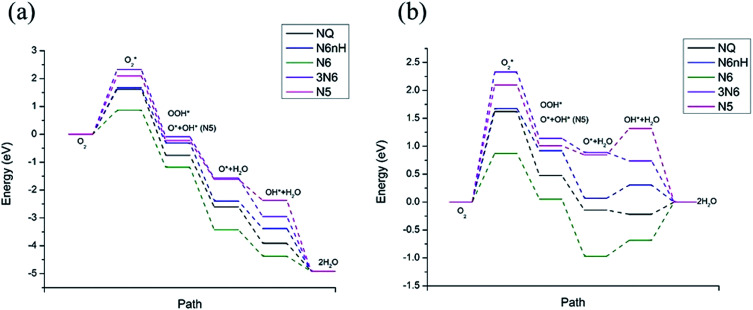
Free energy diagram at (a) *U*_0_ = 0 V (*vs.* NHE) and (b) at *U*_eq_ = 1.23 V (*vs.* NHE). Reaction pathway is O_2_, 
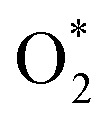
, OOH*, O* + H_2_O, OH* + H_2_O, 2H_2_O.

Fig. 4 shows the free energy diagram and formation energies of each step. We can observe that the first step of oxygen adsorption is uphill for all models, indicating this as the rate-determining step that decides the performance of ORR. All subsequent changes in free energy of the other steps are downhill, meaning that the series of reactions is spontaneous once the initial barrier from oxygen adsorption is overcome. From the free energy diagram, we can conclude that N6 exhibits the lowest overall reaction free energy at *U*_0_, indicating that N6 has the best ORR performance. We can thus rank the ORR reactivity in order of N6 > NQ > N6nH > N5 > 3N6 based on the free energy diagram.

The influence on the ORR pathway of equilibrium potential (*U*_0_), which is the maximum potential of the fuel cell allowed by thermodynamics, have been studied.^39^ In reality, the cathode electrocatalyst for ORR works under external electrode potential. Fig. 4b is a free energy diagram at *U*_eq_ = 1.23 V (*vs.* NHE). We observe that in addition to the initial barrier at step 1 discussed above, N6, N6nH, and N5 need to overcome another barrier at step 4, the adsorption of OH. Furthermore, NQ and N6 need to overcome a barrier at the last step where the second water molecule is formed. The barriers for the adsorption of oxygen are still the same as the zero potential case. The appearance of additional energy barriers might cause the whole reaction to be less active and slow down the reaction, except for 3N6, which still only has one barrier at the first step and spontaneously reacts after overcome that barrier. Hence, we can conclude that the reactivity of all models at equilibrium potential are lower than at zero potential due to the presence of additional energy barriers at equilibrium potential. Nonetheless, N6 still exhibits the lowest overall free energy at equilibrium potential due to its relatively small barrier at the rate-determining step.

**Fig. 5:**
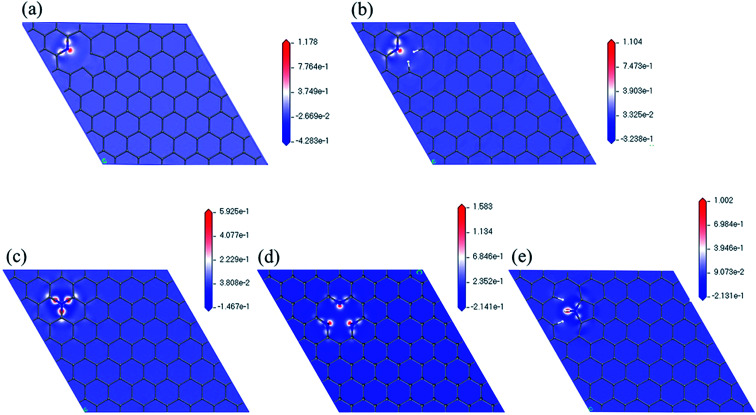
Electron density difference maps with respect to nitrogen atom of (a) N6nH, (b) N6, (c) NQ, (d) 3N6, and (e) N5.

Fig. 5 shows the maps of electron density difference with respective to the nitrogen atoms of the five models. Red indicates regions with higher electron density that are more active ORR sites. We can observe that there are more electrons near nitrogen in pyridinic configuration (N6nH, N6, 3N6). In all five cases, carbon atoms near the nitrogen atom and carbon–nitrogen bonds have higher electron density. This further supports the initial locations for oxygen on nitrogen-doped graphene used in this study.

**Fig. 6:**
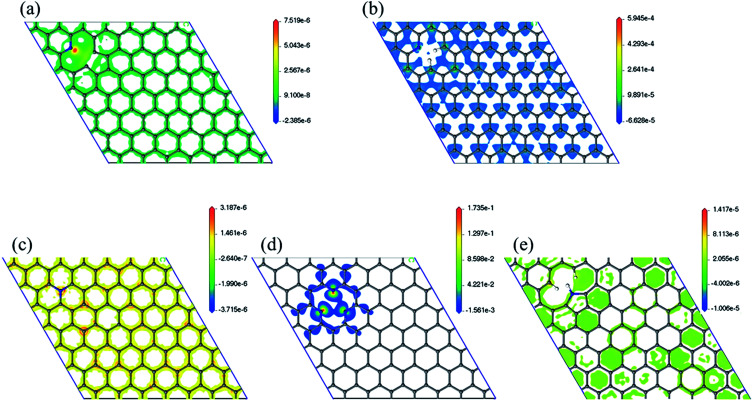
Spin density map with positive values of (a) N6nH, (b) N6, (c) NQ, (d) 3N6, and (e) N5. Mapping with the same scale can be found in the ESI (Fig. S9†).

Spin density has been analyzed in literature as a factor to explain ORR reactivity. Hung considered that neighboring carbons with more spin positive density caused by neighboring nitrogen defects serve as catalytically active sites toward ORR.^23^ From the spin density maps in Fig. 6, we can observe that 3N6 contains sites with the highest spin density value, followed by N6, N5, N6nH, and NQ. In fact, the spin density values of 3N6 are larger than that found in the other systems by several orders of magnitude, and mapping with the same scale cannot sufficiently show the details of the spin density (Fig. S9†). However, 3N6 only has three such positive spin density regions while N5 has more positive spin density but not at the carbon atoms for the most part. NQ, N6nH, and N6 have well distributed high positive spin density regions around carbon atoms although they do not exhibit very high positive spin density values. We consider two aspects of the spin density map that indicate ORR reactivity: (1) regions in the model having the highest positive spin density should also have higher reactivity; and (2) uniform distribution of positive spin density over the material, especially in bonds and atoms, result in more regions having positive spin density and an increased reactivity. With no doping configuration showing both characteristics, we need to consider the combined effects of these aspects to evaluate the reactivity of different nitrogen configurations from spin density analysis. N6 contains several high positive spin density sites within the structure while maintaining a well-distributed spin density, and therefore should be the most ORR active.

## Conclusions

We have successfully simulated the associative mechanisms of ORR for four nitrogen-doped graphene models (N6, N6nH, 3N6, and NQ) and the dissociative mechanism for one model (N5). Energies and optimized structures for each step of the ORR have been calculated to obtain the free energy diagram pathway. Results indicate that N6, which exhibited the lowest overall reaction energy, was the best candidate for ORR in our DFT calculations. Further analysis of charge density maps confirm the initial assumed positions of oxygen on the graphene material. Although we cannot connect spin density map to the free energy diagram directly, it can still provide collaborating results of the reactivity of different nitrogen configurations toward ORR by combining both aspects of calculated spin density map. Analysis shows that N6 is the most active nitrogen-doping configuration of the graphene toward ORR, matching our calculated ORR pathway results.

## Conflicts of interest

There are no conflicts to declare.

## Supplementary Material

RA-009-C8RA08576E-s001
