# Supplementary material for: Effect of nitrogen-doping configuration in graphene on the oxygen reduction reaction
Source: RSC Adv. 2019 Feb 19;9(11):6035–41. doi: 10.1039/c8ra08576e (PMC9060867; doi:10.1039/c8ra08576e)
Supplement: RA-009-C8RA08576E-s001 [file RA-009-C8RA08576E-s001.pdf]

Electronic Supplementary Material (ESI) for RSC Advances.
This journal is © The Royal Society of Chemistry 2018

Electronic Supplementary Information

Effect of Nitrogen-Doping Configuration in Graphene on Oxygen Reduction Reaction

*Shih-Hsuan Tai, Bor Kae Chang**

Department of Chemical & Materials Engineering, National Central University, Zhongli District, Taoyuan City 32001, Taiwan, R.O.C.

*E-mail address: BKChang@ncu.edu.tw

The following figures show the chosen sites of ORR pathway for each step on different models (NQ, N6nH, 3N6, N5), and the structures with the lowest energy in each case.

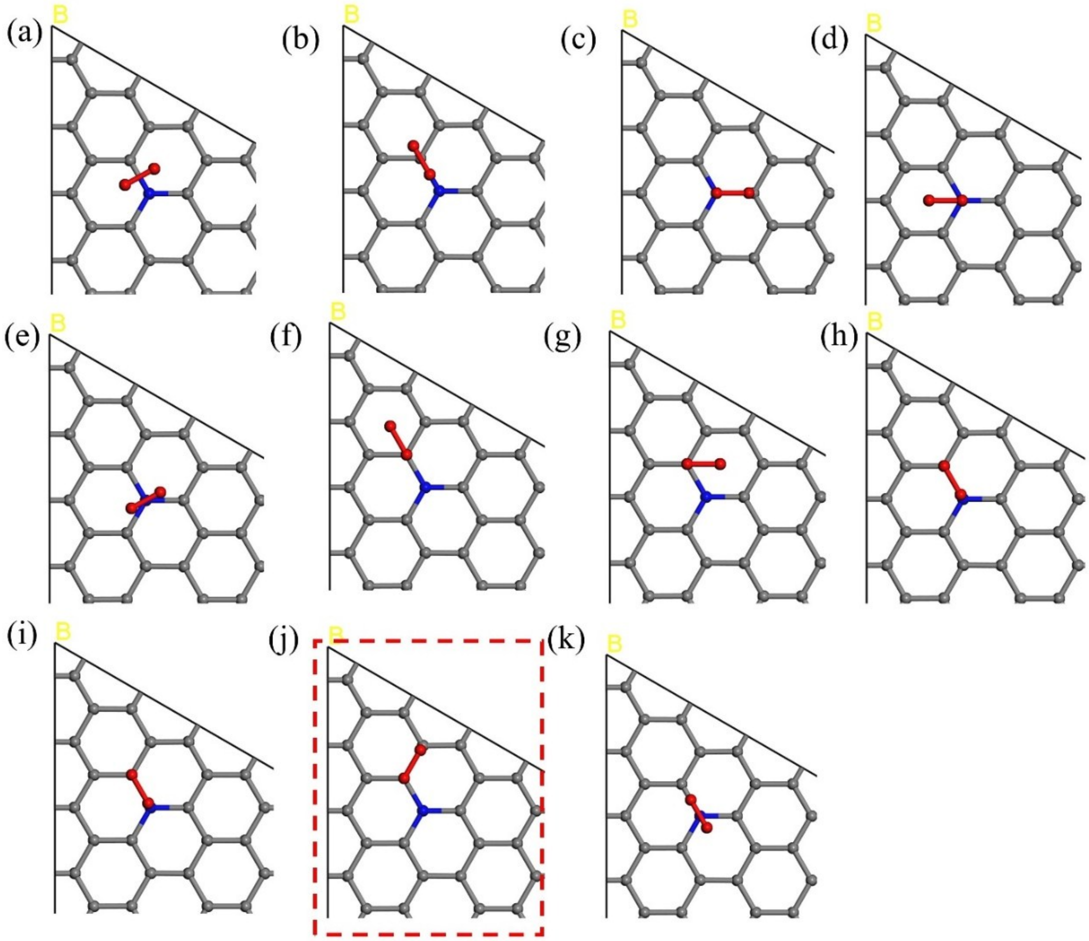

Figure S1. The first step of ORR pathway. (a)-(k) refer to the different sites of O_2 located on NQ substrate. NQ_S1_ (a) HBH (b) BCH (c) NBC (d) NH (e) HCH (f) HNH (g) CH (h) CH2 (i) CBN (j) CBC (k) BNH. The model marked by red dash line is the lowest energy configuration, as discussed in free energy diagram.

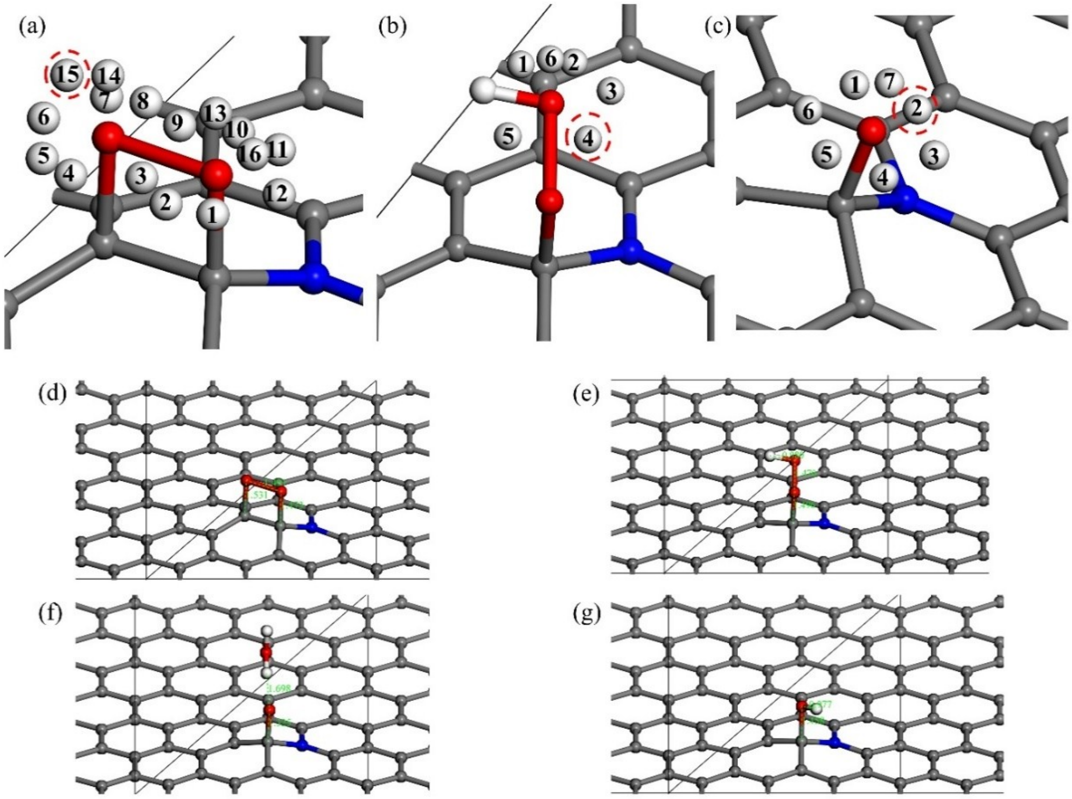

Figure S2. (a)-(c) refer to the chosen sites on NQ for steps 2-4, while (d)-(g) refer to structures with the lowest energy at steps 1-4: (d) NQ_S1_CBC (e) S2_site15 (f) NQ_S3_site2 (g) NQ_S4_site2. The three positions marked by red dash line are the lowest energy configurations, as discussed in the free energy diagram.

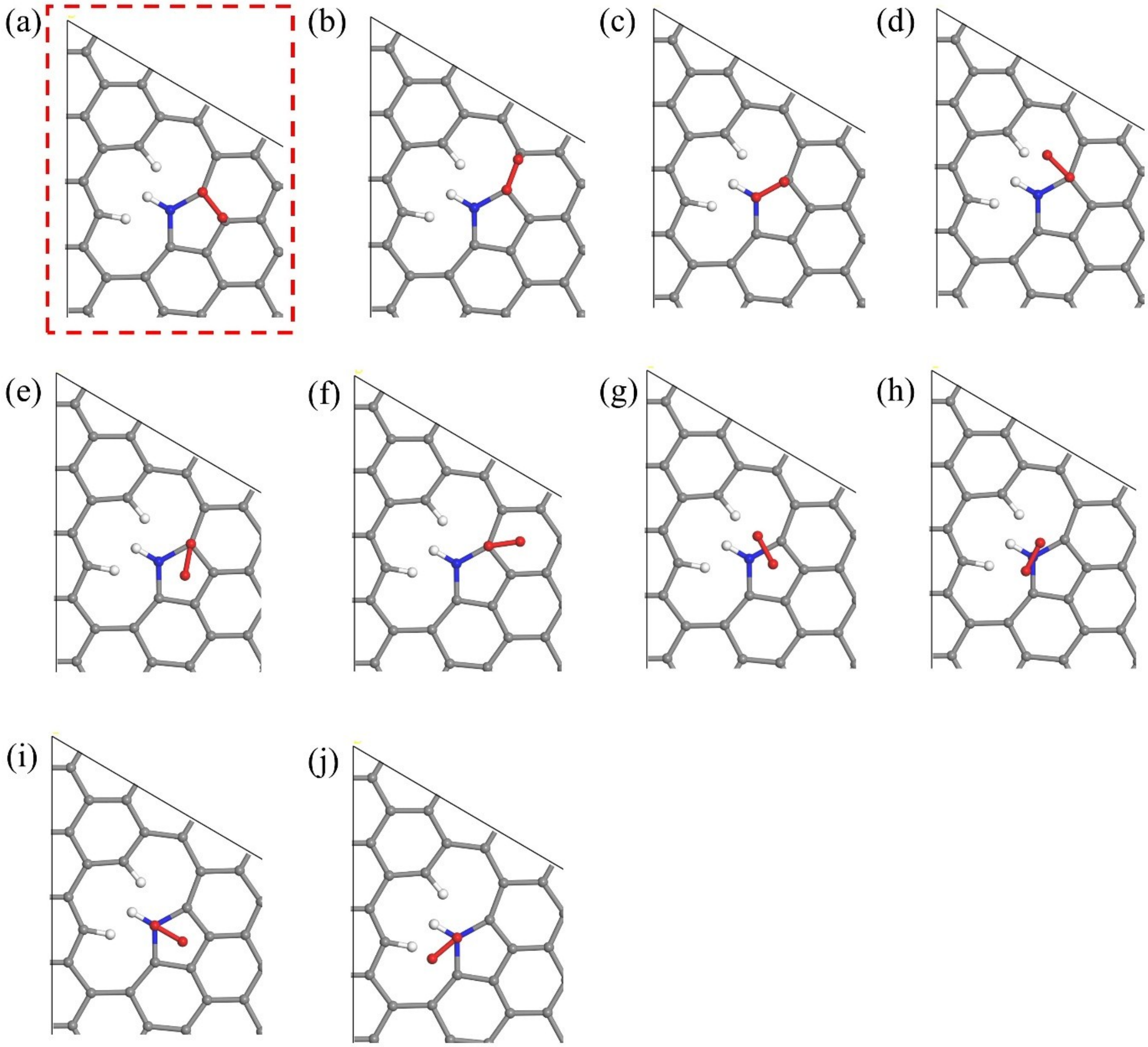

Figure S3. The first step of ORR pathway. (a)-(k) refer to the different sites of O_2 located on N5 substrate. N5_S1_ (a) CBC1 (b) CBC2 (c) CBN (d) CH1 (e) CH2 (f) CH3 (g) HBH (h) HNH (i) NH1 (j) NH2. The model marked by red dash line is the lowest energy configuration, as discussed in the free energy diagram.

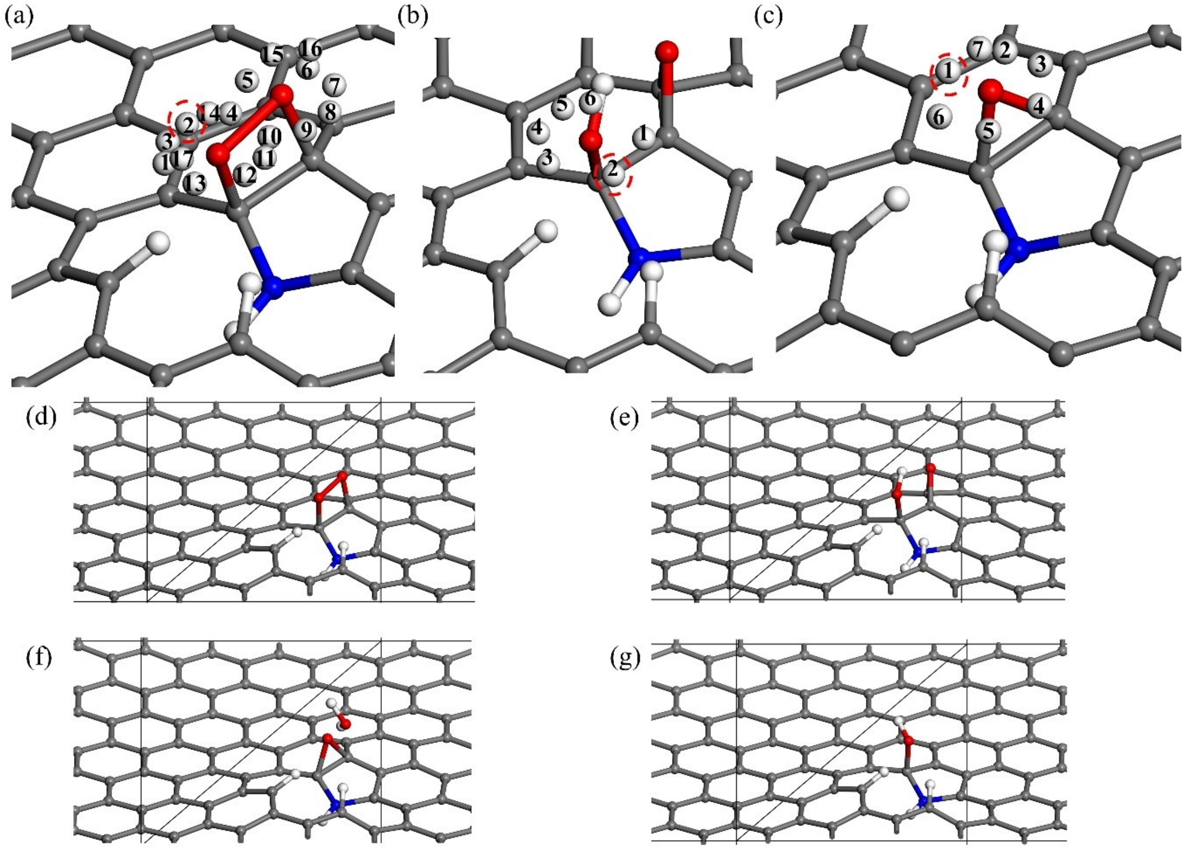

Figure S4. (a)-(c) refer to the chosen sites on N5 for steps 2-4, while (d)-(g) refer to structures with the lowest energy at steps 1-4: (d) N5_S1_CBC1 (e) N5_S2_site2 (f) N5_S3_site2 (g) N5_S4_site1. The three positions marked by red dash line are the lowest energy configurations, as discussed in the free energy diagram.

For the remaining two doped configurations, we followed the procedure for calculating four steps of NQ to compose the free energy diagrams. However, there were still a few differences when choosing sites at each step in different doped configurations due to the symmetry or experience after finishing calculating steps of NQ. Fig. S5 – S8 show all chosen sites at each step and the final structures with the lowest energy in N6nH and 3N6.

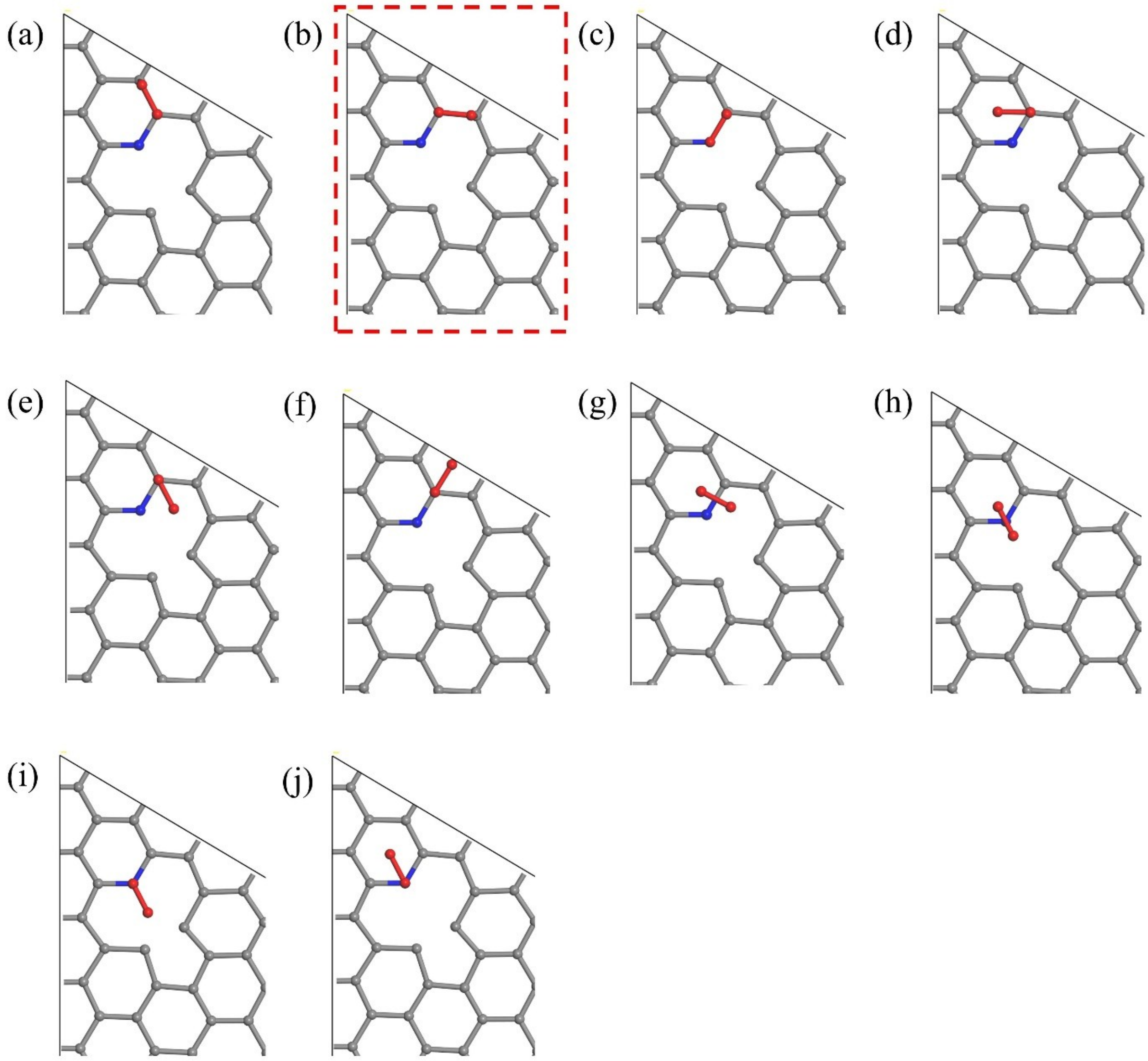

Figure S5. The first step of ORR pathway. (a)-(k) refer to the different sites of O_2 located on N6nH substrate. N6nH_S1_ (a) CBC1 (b) CBC2 (c) CBN (d) CH1 (e) CH2 (f) CH3 (g) HBH (h) HNH (i) NH1 (j) NH2. The model marked by red dash line is the lowest energy configuration, as discussed in the free energy diagram.

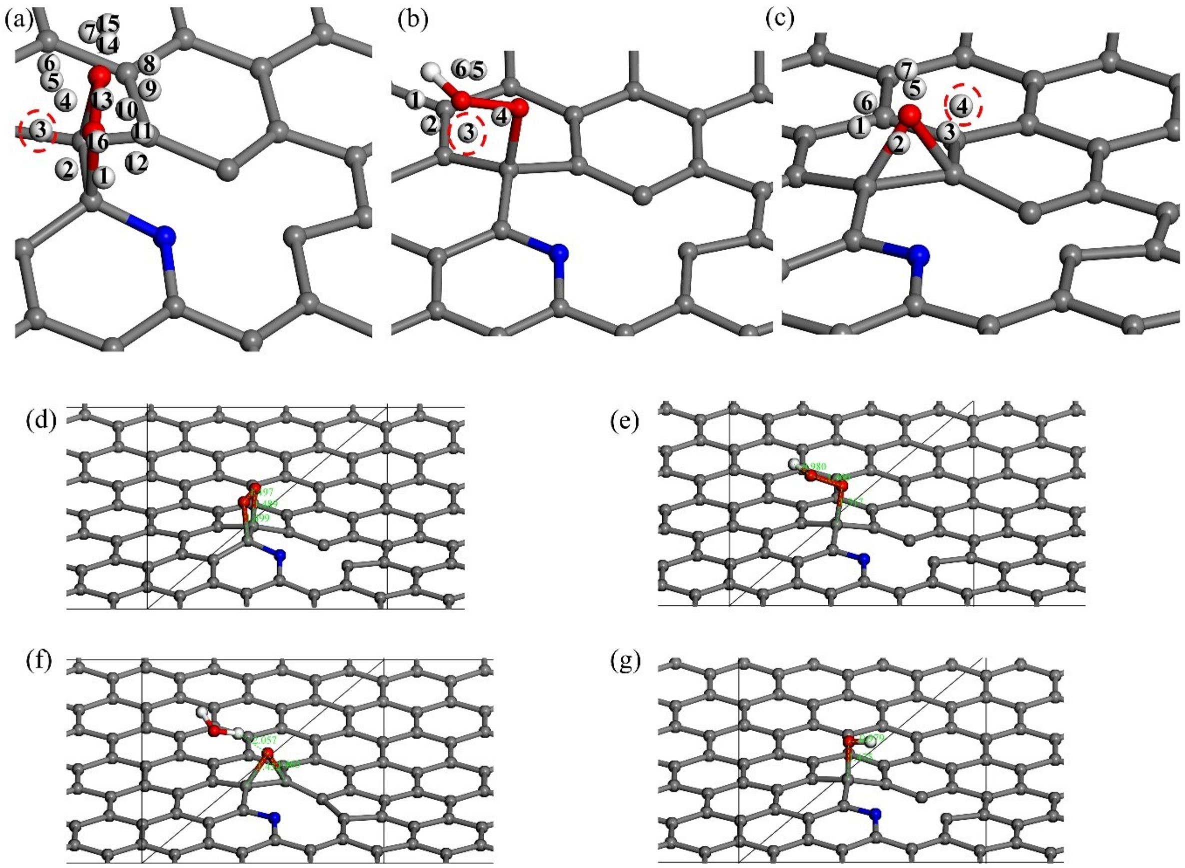

Figure S6. (a)-(c) refer to the chosen sites on N6nH for steps 2-4, while (d)-(g) refer to structures with the lowest energy at steps 1-4: (d) N6nH_S1_CBC2 (e) N6nH_S2_site3 (f) N6nH_S3_site3 (g) N6nH_S4_site4. The three positions marked by red dash line are the lowest energy configurations, as discussed in the free energy diagram.

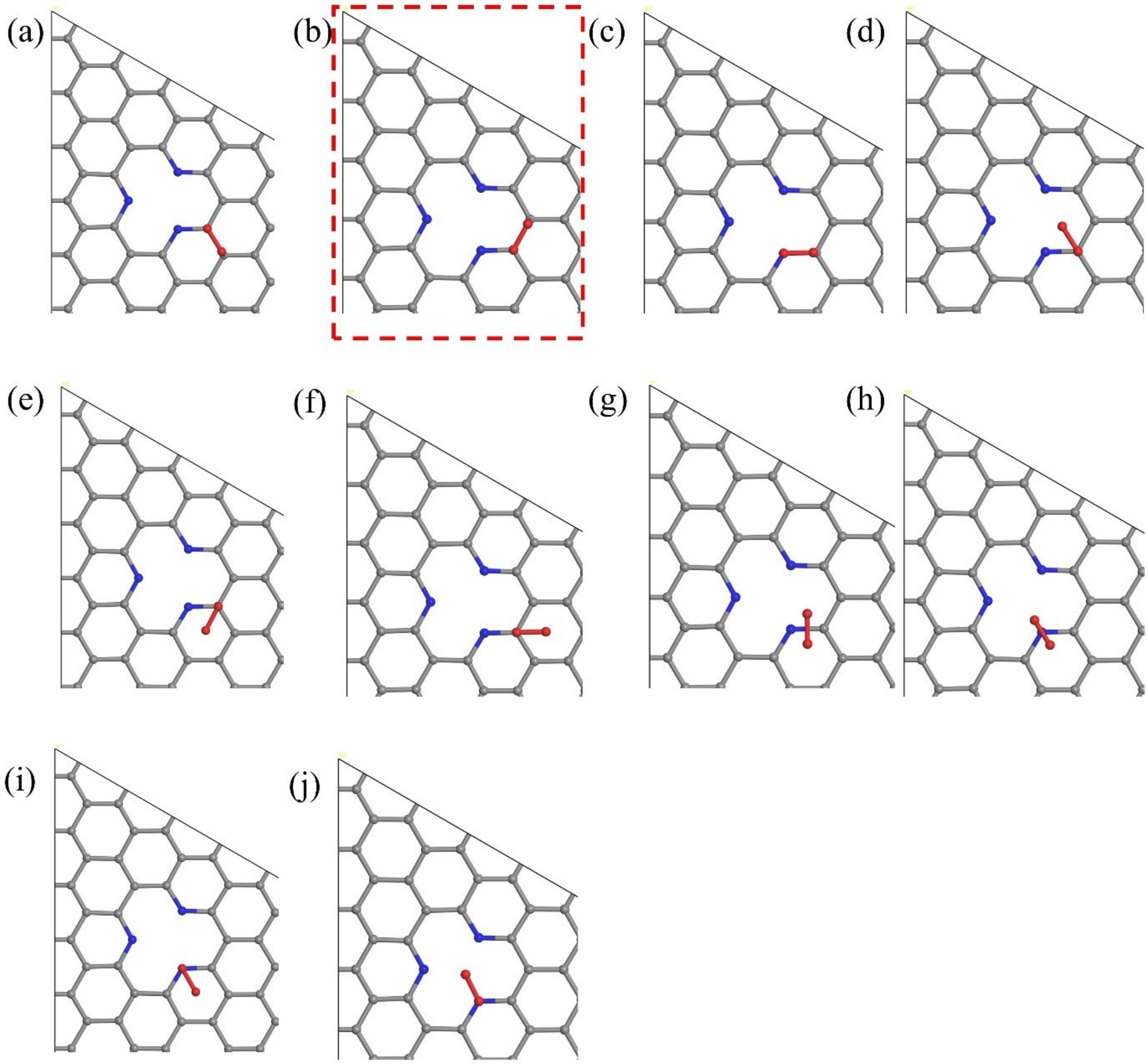

Figure S7. The first step of ORR pathway. (a)-(j) refer to the different sites of O_2 located on 3N6 substrate. 3N6_S1_(a) CBC1 (b) CBC2 (c) CBN (d) CH1 (e) CH2 (f) CH3 (g) HBH (h) HNH (i) NH1 (j) NH2. The model marked by red dash line is the lowest energy configuration, as discussed in the free energy diagram.

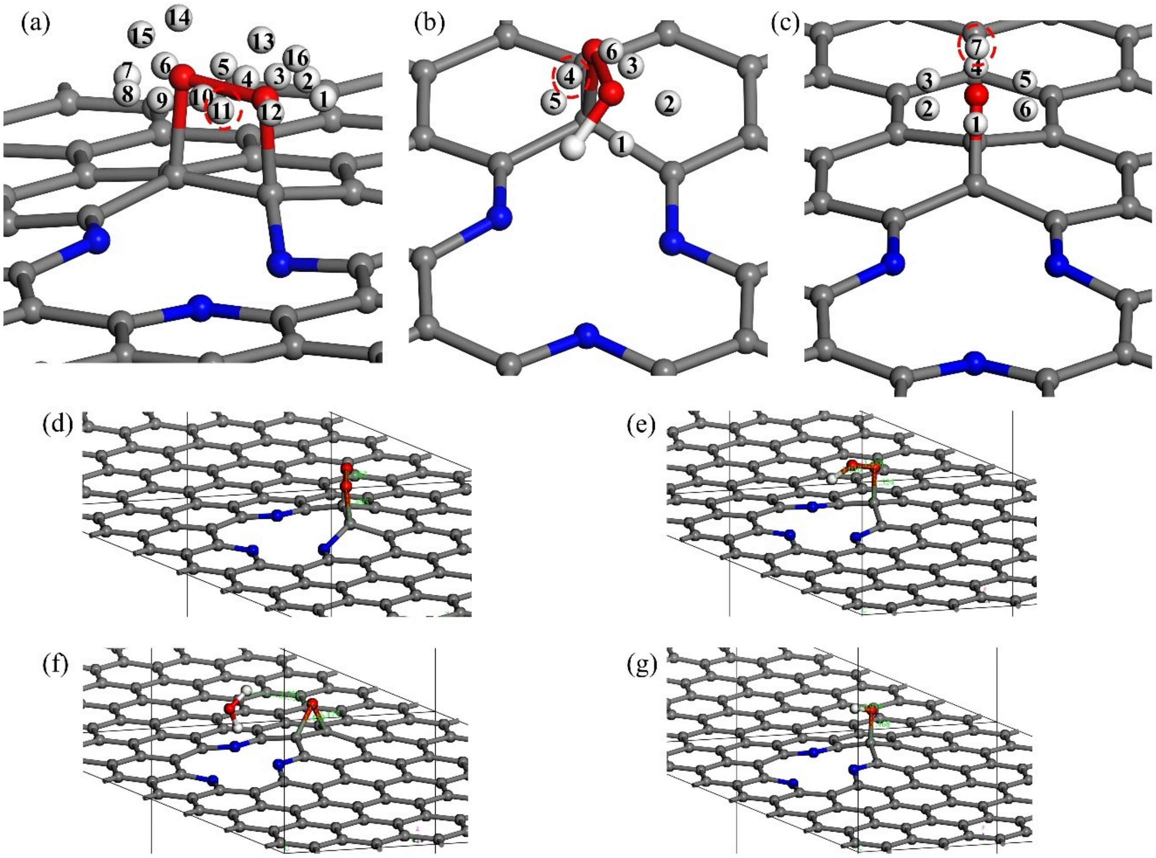

Figure S8. (a)-(c) refer to the chosen sites on 3N6 for steps 2-4, while (d)-(g) refer to structures with the lowest energy at steps 1-4: (d) 3N6_S1_CBC2 (e) 3N6_S2_site11 (f) 3N6_S3_site4 (g) 3N6_S4_site7. The three positions marked by red dash line are the lowest energy configurations, as discussed in the free energy diagram.

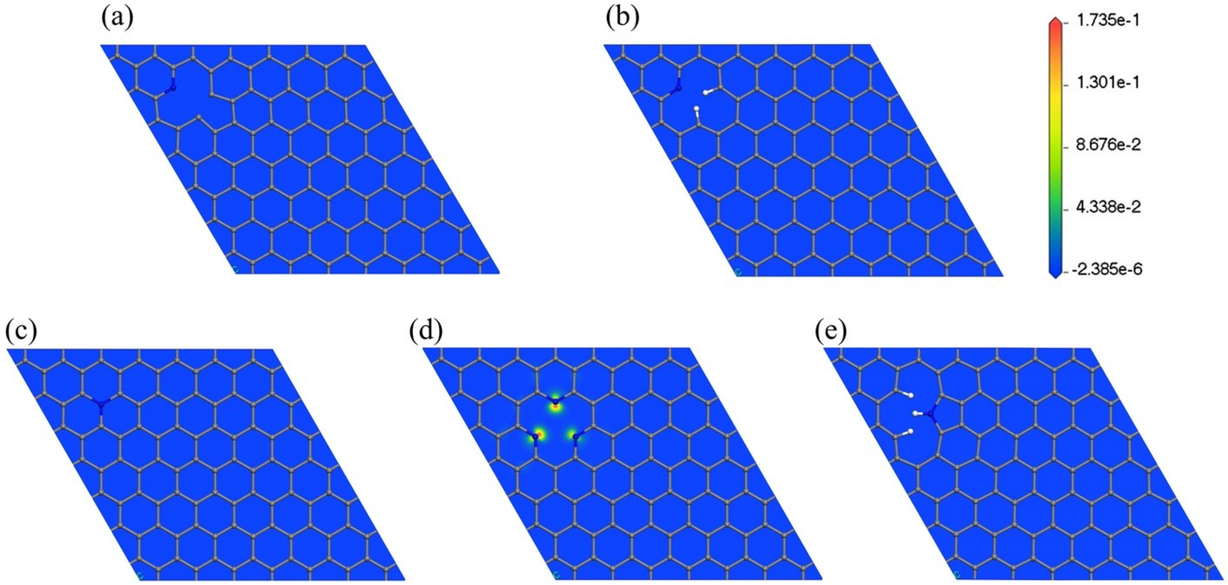

Figure S9. Spin density map of (a) N6nH, (b) N6, (c) NQ, (d) 3N6, and (e) N5 with scale from -2.385e-6 to 1.735e-1.

Table S1. Formation energies (eV) of each reaction pathway at U_0 = 0 V (vs NHE)

Reaction pathway (U_0=0 V)	N6nH	N6	NQ	3N6	N5
1	1.68	0.87	1.62	2.33	2.10
2	-1.99	-2.05	-2.37	-2.42	-2.32
3	-2.08	-2.25	-1.85	-1.48	-1.39
4	-0.99	-0.95	-1.30	-1.38	-0.76
5	-1.54	-0.55	-1.01	-1.97	-2.55

Table S2. Formation energies (eV) of each reaction pathway at U_{eq} = 1.23 V (vs NHE)

Reaction pathway (U_{eq}=1.23 V)	N6nH	N6	NQ	3N6	N5
1	1.68	0.87	1.62	2.33	2.10
2	-0.76	-0.82	-1.14	-1.19	-1.09
3	-0.85	-1.02	-0.62	-0.25	-0.16
4	0.24	0.28	-0.07	-0.15	0.47
5	-0.31	0.68	0.22	-0.74	-1.32
